# Fast Determination of a Novel Iron Chelate Prototype Used as a Fertilizer by Liquid Chromatography Coupled to a Diode Array Detector

**DOI:** 10.1021/acs.jafc.1c05943

**Published:** 2021-12-15

**Authors:** Silvia Valverde, Alejandra Arcas, Sandra López-Rayo, Juan J. Lucena

**Affiliations:** Departamento de Química Agrícola y Bromatología, Universidad Autónoma de Madrid, 28049 Madrid, Spain

**Keywords:** benzeneacetic acid, 2-hydroxy-α-[(2-hydroxyethyl) amino], liquid chromatography, iron deficiency, micronutrients, agronomic efficiency

## Abstract

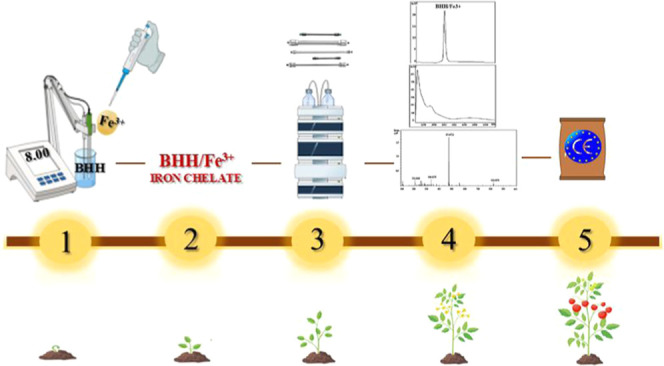

The environmental risk of the application of synthetic chelates has favored the implementation of new biodegradable ligands to correct Fe-deficient plants. This study developed and validated an analytical method for determination of a new prototype iron chelate—Fe(III)-benzeneacetate, 2-hydroxy-α-[(2-hydroxyethyl)amino]—(BHH/Fe^3+^) based on liquid chromatography with diode array detection, as a potential sustainable alternative. Chromatographic analysis was performed on a LiChrospher RP-18 in reverse-phase mode, with a mobile phase consisting of a mixture of acetonitrile (solvent A) and sodium borate buffer 0.20 mM at pH = 8 (solvent B) at a flow rate of 1.0 mL/min in isocratic elution mode. This method was fully validated and found to be linear from the limit of quantification (LOQ) to 50 mg/L and precise (standard deviation below 5%). The proposed method was demonstrated to be selective, precise, and robust. The developed methodology indicated that it is suitable for the quantification of iron chelate BHH/Fe^3+^.

## 1 Introduction

Currently, the most effective method for curing iron deficiency in crops is the application of iron fertilizers to soil or foliage. Iron fertilizers must comply with current EU regulations EU2003/2003 (EU Directive, 2003, and subsequent amendments) and EU 1009/2019.^1,2^ EU2003/2003 includes FeSO_4_ as the only Fe^2+^ inorganic salt, synthetic Fe^3+^ chelates, and a selected number of Fe complexes of low stability.^1^ Inorganic salts have low efficiency in neutral–basic soils due to their rapid precipitation, and thus their use is limited to low reactive media or foliar applications. Synthetic iron chelates, which are widely used in agriculture, are products of medium–high stability using polyaminocarboxylate chelating agents. Chelates are complex organic molecules in which Fe^3+^ is surrounded by a coordination sphere formed by chelating agents such as organic anions that are able to donate electrons to the metal center. This prevents metal precipitation, and the iron remains in solution and is transported to the plant root.^3,4^ Among the most commonly used chelating agents are ethylenediaminetetraacetic acid (EDTA), ethylenediamine-*N*-*N*′bis(*o*-hydroxyphenylacetic) acid (*o*,*o*-EDDHA), and *N*-*N*′bis(*o*-hydroxyphenyl) ethylenediamine-*N*-*N*′-diacetic acid (HBED) with medium-to-high affinity to Fe.^5^ These synthetic chelates are agronomically efficient and generally persistent in the environment,^6,7^ thus presenting environmental risks.^8^ Consequently, there is considerable interest now in finding new degradable iron chelates that are effective but have a lesser environmental impact than traditional synthetic chelates.^9,10^

**Figure 1:**
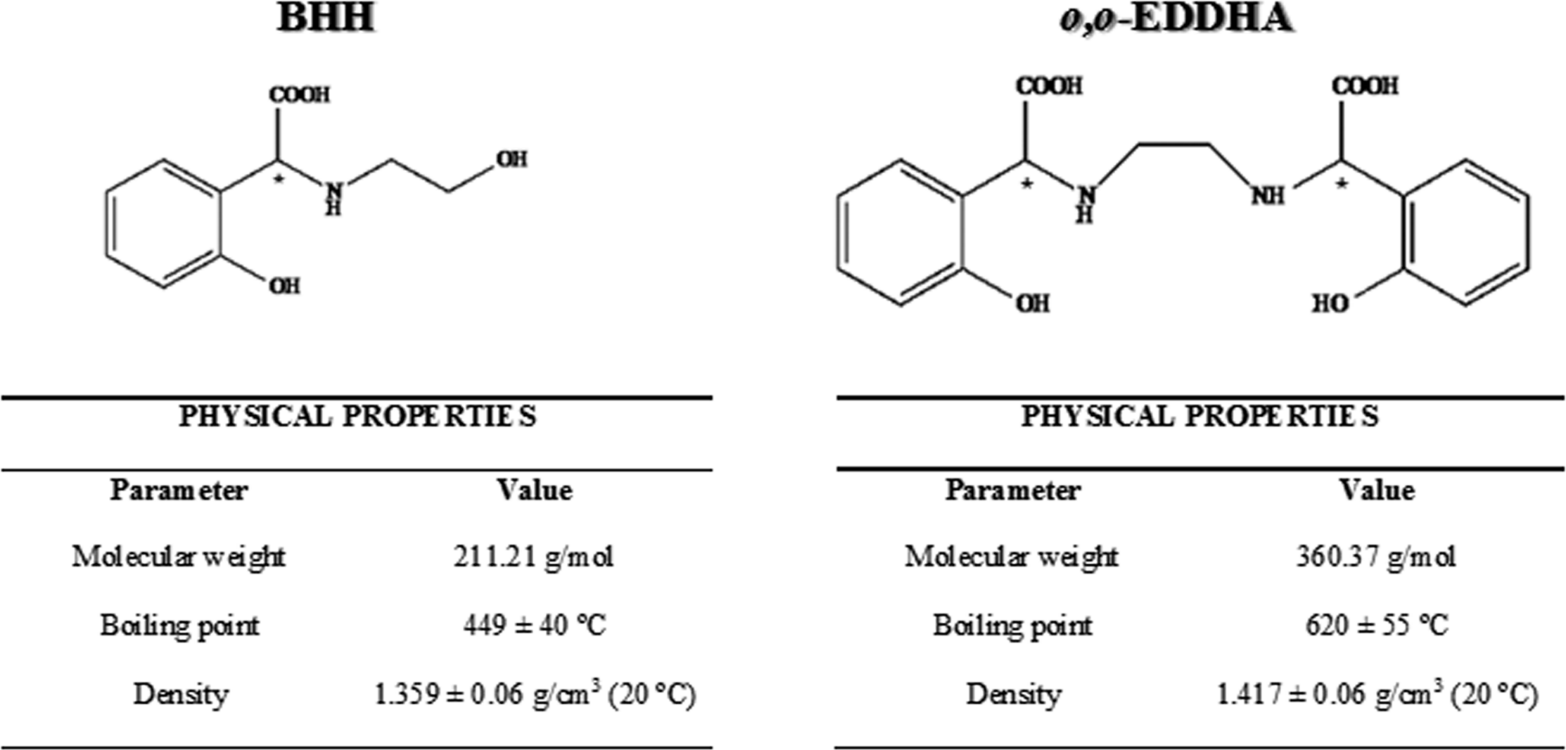
Chemical structure and main physicochemical properties of BHH (C_10_H_13_NO_4_; molecular weight, 211.21) and *o*,*o*-EDDHA (C_18_H_20_N_2_O_6_; molecular weight, 360.37). *Denotes asymmetric carbons.

This study focused on a potential new iron chelate based on the benzeneacetic acid, 2-hydroxy-α-[(2-hydroxyethyl)amino] (BHH) chelating agent (for its structure and main physicochemical properties, see Figure 1) whose Fe-chelated content in commercial formulations may reach 8% (w/w). The ligand has a secondary amine, two hydroxyl groups—one of them phenolic, the other carboxyl—and one chiral carbon; thus it may occur as two possible isomers S and R. Its structure can be compared with the chelating agent *o*,*o*-EDDHA (Figure 1), which presents two secondary amines and two chiral carbons. While *o*,*o*-EDDHA forms hexadentate complexes, BHH has lower coordination, allowing an open structure of the chelate and making the Fe-chelate union more accessible. Its stability is expected to be lower, and it will gradually degrade, providing iron to the plant, as a sustainable chelating agent for use as a ferric chelate. Its effectiveness in agronomic conditions is expected to be comparable to *o*,*o*-EDDHA due to their structural similarity.

Liquid chromatography (LC) using C_18_^11−21^-based^11−21^ analytical columns is the technique of choice for determining iron chelates in solution or commercial products, in view of the existing literature and European legislation on the use of iron chelates as fertilizers, as indicated by the European Committee for Standardization (CEN). Moreover, in recent years, the coupling of LC with mass spectrometry (MS),^14,20^ especially tandem mass spectrometry (MS/MS) using the electrospray (ESI) source in negative mode ionization in most cases^17−21^ and atmospheric pressure chemical ionization in some studies,^13,14^ has become one of the preferred analytical techniques for analyzing metal-chelator complexes^13,17,18,20,21^ due to its sensitivity and selectivity. Nonetheless, diode array detectors (DAD) have been employed extensively in many studies^11,12,15,16,20^ and routine laboratories because they are affordable and reliable detectors.

The aim of this study was to propose, for the first time, a specific analytical methodology to quantify BHH/Fe^3+^ by LC-DAD and confirmed by MS/MS. Retention and separation using hydrophilic interaction liquid chromatography (HILIC) and reverse-phase liquid chromatography (RPLC) were compared.

It was consequently determined that separation would be carried out using a LiChrospher RP-18. The effects of various parameters were studied, such as mobile-phase composition, pH, the type of organic modifier, the influence of addition of different additives and flow rate. A further goal of the present study was to perform a complete validation of the proposed method to determine BHH/Fe^3+^ in the potential commercial product. To approve the inclusion of a fertilizer in the list of authorized compounds of the UE Regulation, an analytical method approved by CEN able to determine the chelated Fe and/or the ligand content is necessary.

## 2 Materials and Methods

### 2.1 Reagents

Sodium hydroxide (NaOH) and iron (III) nitrate nonahydrate (Fe (NO_3_)_3_·9H_2_O) were obtained from Merck KGaA (Darmstadt, Germany) and hydrochloric acid (HCl) was obtained from PanReac (Barcelona, Spain). LC-grade ethanol (EtOH), methanol (MeOH), and acetonitrile (ACN) were supplied by Scharlau Chemie S.A. (Barcelona, Spain). Formic acid, acetic acid, boric acid, ammonium formate, ammonium acetate, sodium formate, ammonium monobasic dihydrogen phosphate, ammonium dibasic monohydrogen phosphate, ammonium bicarbonate, trisodium citrate, sodium borate, diethylamine (DEA), triethylamine (TEA), 2-amino-2-hydroxymethyl-propane-1,3-diol (TRIS), and 2-(*N*-morpholino) ethanesulfonic acid (MES) were obtained from Sigma-Aldrich Chemie GmbH (Steinheim, Germany). Ammonium hydroxide (NH_4_OH) was purchased from Scharlau Chemie S.A. (Barcelona, Spain). Tetrabutylammonium hydroxide (40% solution in water) was supplied by Sigma-Aldrich (Darmstadt, Germany). All of the chemicals used were of analytical grade. Syringe filters (17 mm, nylon 0.45 μm) were purchased from Labbox Labware S.L. (Barcelona, Spain) and ultrapure water was obtained using Millipore Milli-RO plus and Milli-Q systems (Bedford, MA).

### 2.2 Standard Solutions

The standard chelating agent (BHH) and a sample prototype of BHH/Fe^3+^ were obtained as described by Vicente and Blasco.^22^ The titrimetric purity of the chelating agent determined using a photometric method^4^ was 84.6 ± 0.5%.

Briefly, about 1.0 × 10^–4^ M ligand solution was titrated with a 4.48 × 10^–4^ M Fe(III) standard solution (Fe(NO_3_)_3_ in HNO_3_ 0.5 mol/L) provided by Merck KGaA (Darmstadt, Germany) until absorbance at 480 nm presented no changes. Titration was carried out at 25.0 ± 0.5 °C in a sealed, water-jacked glass vessel and in purified N_2_ atmosphere, and iron was added with a 721 NET Titrino potentiometric titrator (Metrohm AG, Herisau, Switzerland). Ionic strength was maintained at 0.1 M with NaCl, and pH was fixed at 6.0 with 2 mM MES controlled by a pH-Stat system (Metrohm AG, Herisau, Switzerland).

To prepare BHH/Fe^3+^ standard solution, the ligand was dissolved in NaOH (ligand/NaOH, 1:3 molar ratio). An amount of Fe (NO_3_)_3_·9H_2_O, calculated to be 5% in excess of the molar amount of the ligand, was added while keeping the solution pH in the range of 6–8 with NaOH or HCl. The solution pH was adjusted to 8.0 at the end of the iron addition and left to stand overnight to allow excess Fe to precipitate as oxyhydroxides. It was then filtered through a 0.45 μm Millipore cellulose membrane and made up to volume with water. The Fe concentration in the final solution was assessed by atomic absorption spectrophotometry. This solution was diluted as required.

A solution of the sample prototype (100 mg/L Fe) was prepared by dissolving the formulation (8% Fe) in water and filtering it through a 0.45 μm Millipore cellulose membrane prior to LC analysis. Light exposure was avoided during preparation and storage due to the potential photodecomposition of chelates.^23^

### 2.3 Chromatography Systems

#### 2.3.1 High-Performance Liquid Chromatography (HPLC) Diode Array Detectors (DAD)

Chromatographic analyses were performed on a 1260 Infinity HPLC system (Agilent Technologies, Waldbronn, Germany). The system consisted of an online vacuum degasser, a quaternary pump, a thermostated column compartment and a ultraviolet–visible (UV–vis) detector with variable wavelengths. OpenLAB CDS Rev. C.01.05 v.37 software was used for system control and data acquisition. Different analytical columns used for HPLC studies were tested. RPLC columns: Symmetry *C*_18_ (150 × 3.9 mm^2^; particle size 5 μm), Spherisorb ODS2 C_18_ (250 × 4.6 mm^2^; particle size 5 μm) from Waters (Milford MA), Luna C_18_ (150 × 3.9 mm^2^; particle size 5 μm) provided by Phenomenex (Torrance), LiChrospher RP-18 (150 × 4.6 mm^2^; particle size 5 μm), and a HILIC column, SeQuant ZIC-HILIC (150 × 3.9 mm^2^; particle size 5 μm) were purchased from Merck KGaA (Darmstadt, Germany).

After several optimization studies, LiChrospher RP-18 was chosen as the preferred option due to its better chromatographic performance with the iron chelate investigated. The mobile phase selected was composed of a mixture of acetonitrile and sodium borate buffer 0.20 mM (pH = 8) (70:30, v/v) applied at a flow rate of 1.0 mL/min in isocratic mode. The injection volume was set at 10 μL. Finally, measurements were performed at a wavelength of 250 nm after previously examining the corresponding UV–vis spectra in a spectrophotometer (Figure S1A).

#### 2.3.2 Direct-Infusion MS Analysis

Direct-infusion MS analyses (without column separation) just for confirmatory purposes were performed using a UPLC system (ACQUITY, Waters, Milford, MA) and a QTOF mass spectrometer (maXis impact, Bruker Daltonik GmbH, Bremen Germany) that were coupled through an electrospray (ESI) interface. The sample of BHH/Fe^3+^ was directly injected into the ESI source using a Hamilton syringe and a syringe pump with a flow rate of 3 μL min^–1^ and injection volume of 2.0 μL. The direct-infusion solvent was a mixture of MeOH/H_2_O (v/v, 98:2) solution. Detection conditions using the electrospray (ESI) source in the negative ionization mode were set as follows: capillary voltage 3400 V, drying gas (N_2_) flow 4 L/min, drying gas (N_2_) temperature 200 °C, and nebulizer pressure 0.4 bar. The *m*/*z* scale of the mass spectra was calibrated daily by infusing an atrazine mixture. Spectra were acquired in a mass range of 50–1000 *m*/*z*. The compound showed an intense [M–H]^−^ (precursor ion: 474.0733) to obtain product ions for MS/MS carried out using an isolation width of 5 *m*/*z* and a collision energy of 30 eV. Identification was performed by means of product ions that provided the highest signals. Data were acquired and processed using software Data Analysis 4.4 and Qualitative Analysis from Bruker Daltonik.

## 3 Results and Discussion

### 3.1 Optimizing LC-DAD Conditions

The first studies were dedicated to selecting the most suitable stationary phase to determine BHH/Fe^3+^. Five different types of packing materials were tested. The preliminary studies revealed that the pH of the mobile phase was a critical point and had to be fixed to maintain BHH/Fe^3+^ in the ferrated ligand. The optimal value was found at pH 8; higher pH values could lead to decomplexing or hydroxylation of the iron chelate and lower pH values could lead to the protonation and decomplexing of the chelate or the presence of the protonated Fe^3+^ form (data not shown).

The main characteristics of the analytical columns supplied by the manufacturer are summarized in Table S1.

One of the first packing materials assayed was the hydrophilic interaction liquid chromatography (HILIC) column (SeQuant ZIC-3.5 μm HILIC 150 × 3.9 mm^2^; Merck) due to the emphasis in research of the use of this type of chromatography to separate polar compounds. Several studies were carried out to evaluate the effect of mobile-phase composition in HILIC. Mobile phases composed of aqueous ACN solvents and soluble buffer salts are recommended as they influenced the peak quality.^19^ Among the buffers tested were ammonium formate (pH = 7.5), ammonium acetate (pH = 7.5), and ammonium bicarbonate (pH = 8.5). The use of ammonium salts provided suitable peak shape; however, after examining the UV–vis spectra, the band of the Fe–phenol bonding (around 480 nm) was not observed (data not shown). This event proved that the complex breaks down and that the HILIC separation mechanism was not suitable for this compound.

**Figure 2:**
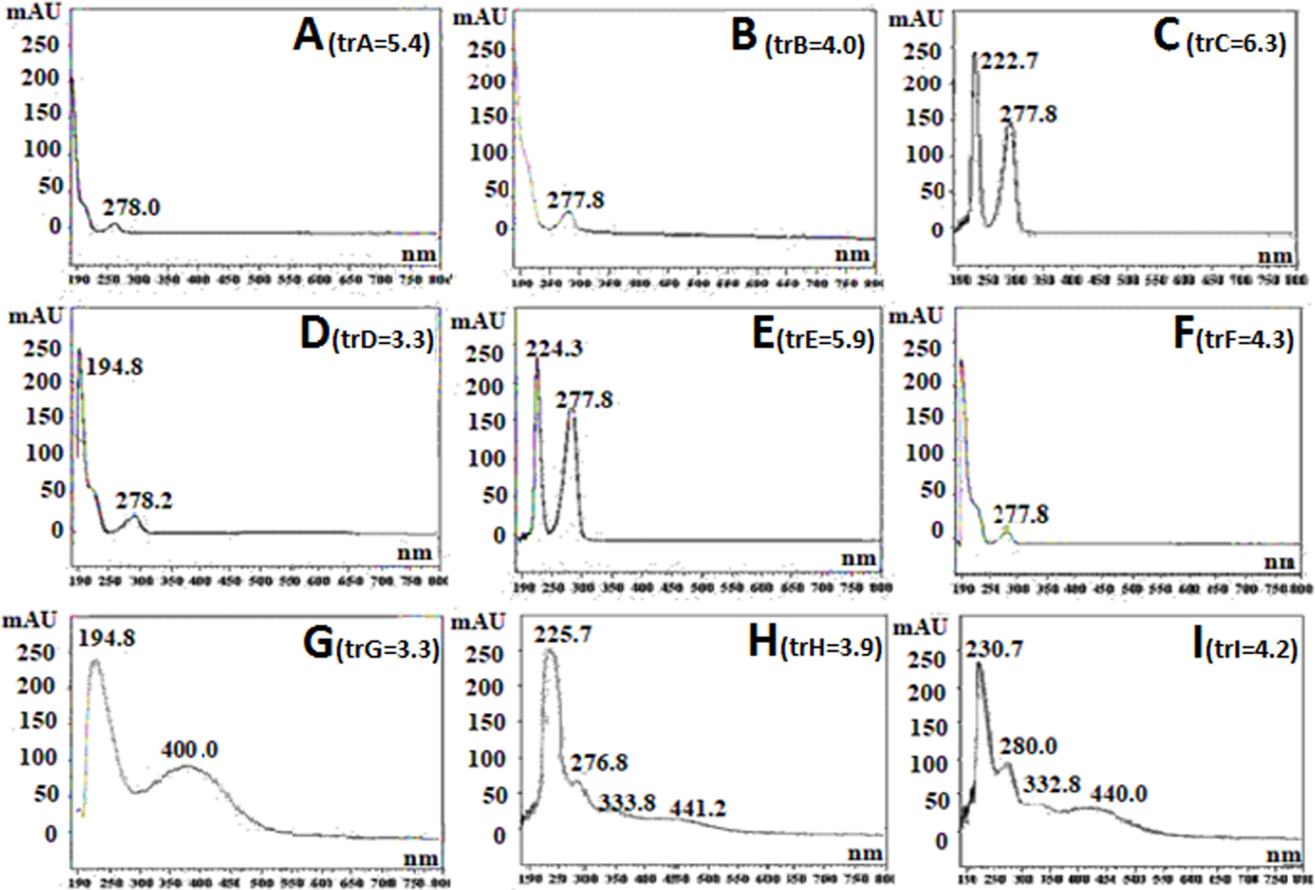
UV–vis spectra obtained for the main peak after testing the following mobile phases: (A) ACN/H_2_O (phosphate buffer, 10 mM, pH = 8), 10:90, v/v; (B) ACN/H_2_O (borate buffer, 10 mM, pH = 8), 10:90, v/v; (C) ACN/H_2_O (ammonium acetate, 20 mM, pH = 7), 10:90, v/v; (D) ACN/H_2_O (ammonium bicarbonate, 10 mM, pH = 8), 10:90, v/v; (E) ACN/H_2_O (sodium formate, 10 mM, pH = 7.5), 10:90, v/v; (F) ACN/H_2_O (Tris–HCl, 10 mM, pH = 8), 10:90, v/v; (G) ACN/H_2_O (trisodium citrate, 10 mM, pH = 8), 5:95, v/v; (H) MeOH/H_2_O (trisodium citrate, 10 mM, pH = 8), 5:95, v/v; and (I) MeOH/H_2_O (trisodium citrate, 50 mM, pH = 8), 5:95, v/v.

Taking into account the structural similarity that this chelate presents compared with *o*,*o*-EDDHA/Fe^3+^, it was decided that different C_18_ columns used in the official methods would be tested.^24,25^ Symmetry *C*_18_ (150 × 3.9 mm^2^; particle size 5 μm) composed of high-purity base-deactivated silica and based on spherical particles and Spherisorb ODS2 C_18_ (250 × 4.6 mm^2^; particle size 5 μm) with a reverse-phase sorbent based on spherical silica particles were tested using established chromatographic methods. As expected, the results showed a loss of symmetry and irreproducible peak because the iron chelate studied is stable at pH 8 and the pHs of the mobile phases tested were lower (pH 6^24^ and pH 3^25^). The ionization state of the analyte directly affects the degree of its interaction with the stationary phase. At these pH levels, the analyte is ionized, more polar, and therefore more likely to participate through hydrogen bonding. In the reversed phase, the analyte will be retained for less time in hydrophobic interactions with the stationary phase and for more time forming hydrogen bonds with the aqueous part of the mobile phase compared with the neutral molecule, providing less retention of the polar analytes. The pH of the mobile phase influences the interactions (hydrophobic, electrostatic, π···π, etc.) that might take place during the chromatographic separation process, so the pH and ionic strength were evaluated. To obtain shorter analysis times, Symmetry *C*_18_ (150 × 3.9 mm^2^; particle size 5 μm) was selected for the optimization experiments. The pH range studied was 7–9, which corresponds to the optimum iron chelate pH and is within the optimal pH of the column. Several experiments varying in the organic solvent and percentage, salts, and concentration were performed (compositions with the best performances in terms of peak shape are summarized in Table S2). When using an eluent with pH lower than 8 (Figure 2C,E), the UV–vis spectrum obtained for the main peak corresponded to that of the free ligand BHH (Figure S1B), certainly because under these conditions the iron complex breaks down. When the pH was adjusted to 8, similar spectra were obtained in all cases (Figure 2A,B,D,F). The saturated band at 225 nm is assigned to the benzene ring of the BHH. The band around 280 nm is typical of the n−π* transitions of C=O groups or π–π* transitions of C=C groups and is ascribed to carbonyl groups or phenolate, respectively, that are present in the structure of BHH.^26−28^ Nevertheless, the band at 480 nm characteristic of the Fe–phenol bonding was not presented, indicating that iron was released, and the complex was not observed. The results obtained with sodium citrate in the mobile phase provided another type of spectra. When ACN was selected as an organic modifier (Figure 2G), the spectrum obtained showed a new intense band around 330 nm, which may correspond to the OH in *ortho* substitution or even to the alcohol–Fe interaction, while the absorbance of the band corresponding to the union Fe-phenolate was low. In the case of MeOH as the organic solvent (Figure 2H,I), the spectra obtained were similar to the spectrum obtained for the BHH/Fe^3+^ standard solution in a spectrophotometer (Figure S1A). Under these chromatographic conditions, a band shift at 440 nm was identified, suggesting that the chelate structure was being modified. This effect can be explained by the complexing capacity of sodium citrate. A competition between ligand BHH and citrate for Fe^3+^ may take place, forming an iron-citrate complex^28^ or a Fe-citrate-BHH chelate. Therefore, the solvent conditions strongly affected Fe complexation during separation. The obtained results were not adequate since in all cases the characteristic band at 480 nm of the Fe-phenolate was not observed and other unknown peaks also appeared, suggesting that the complex broke down or transformed and was therefore not retained.

Thus, it was decided that another packing material would be tested, Luna C_18_ (150 × 3.9 mm^2^; particle size 5 μm), based on porous silica, which has a high surface concentration of silanol groups and spherical particles. In this case, the retention of the iron complex was achieved, providing a single peak with the band at 480 nm, but most of the mobile phase tested provided an excessive peak tailing and very short retention times in all cases (data not shown). It should be noted that this column is suitable for hydrophobic compounds even though it is not suitable for this analyte. It was studied to compare different packaging materials.

**Figure 3:**
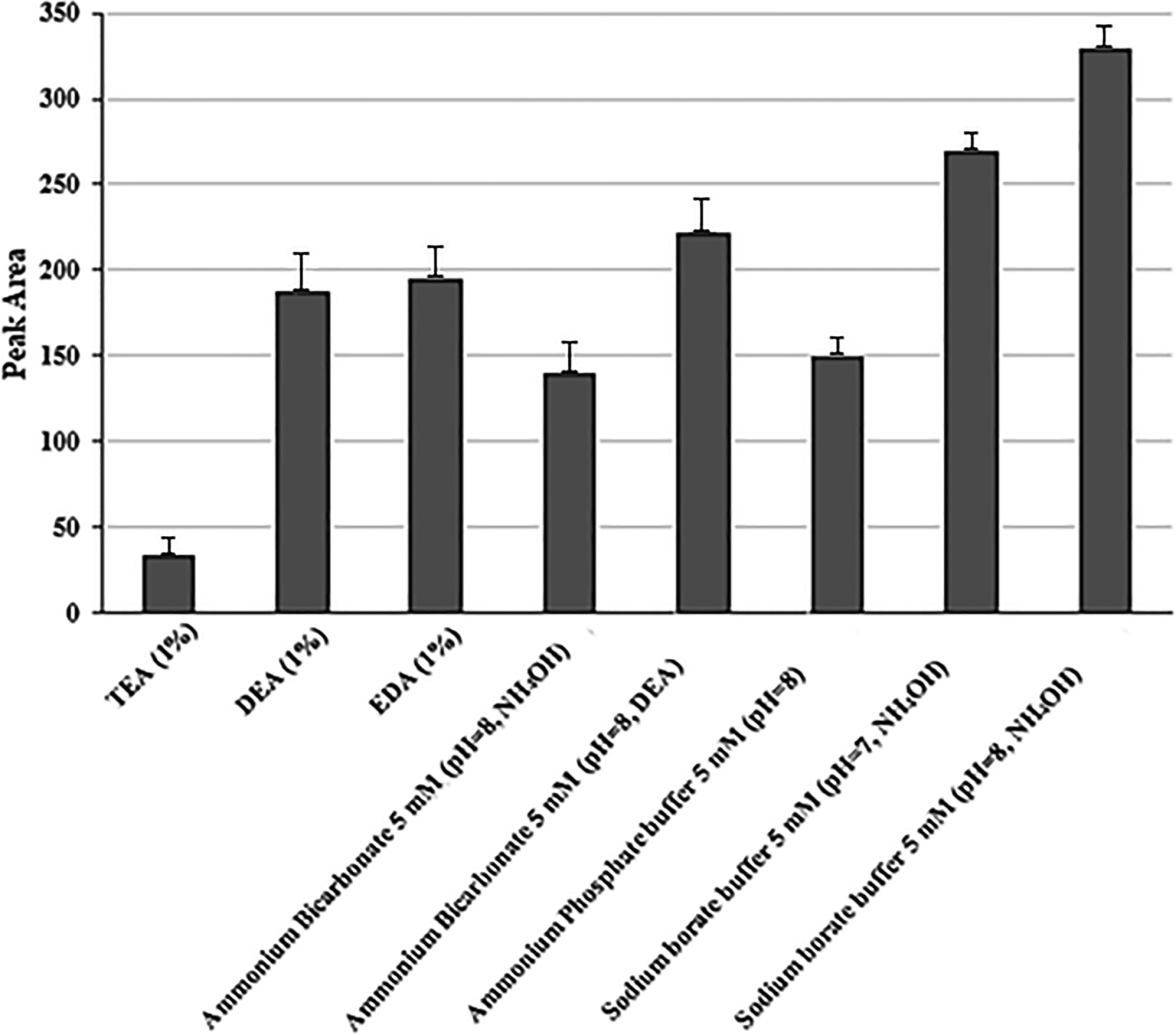
Peak area obtained after testing different mobile phases (*n* = 3) based on the ACN/aqueous solvent (30:70, v/v) at medium QC (10 mg/L Fe).

Finally, the chromatography behavior of LiChrospher RP-18 (150 × 4.6 mm^2^; particle size 5 μm) was studied. This column is made from another type of silica (silica A) with a high number of unprotected silanol groups and adequate for retention of weakly basic compounds. To optimize the organic solvent and its percentage, several experiments were conducted with diverse mobile phases composed of aqueous mixtures of MeOH and ACN. The best results in terms of resolution and analysis time were obtained with the mixture ACN/H_2_O (30:70, v/v). However, peak tailing and pH shifts were observed, so additives were tested to solve it. Different experiments (Figure 3) were performed maintaining the ratio (30:70, v/v) with different bases (DEA, EDA, and TEA) and salts (ammonium bicarbonate, ammonium phosphate, and sodium borate buffers). Successful retention of the complex was achieved in all tests, confirmed by the band at 480 nm. The main difference in this column was the amount of unprotected silanol groups facilitating retention. The highest peak area (Figure 3) was obtained with sodium borate buffer at pH = 8. The influence of concentration (0.1–10 mM) on the separation was studied, and a decrease in the peak area was observed when the concentration increased to 0.5 mM. Thus, 0.2 mM was selected as the optimal sodium borate buffer concentration (see Figure S2).

**Figure 4:**
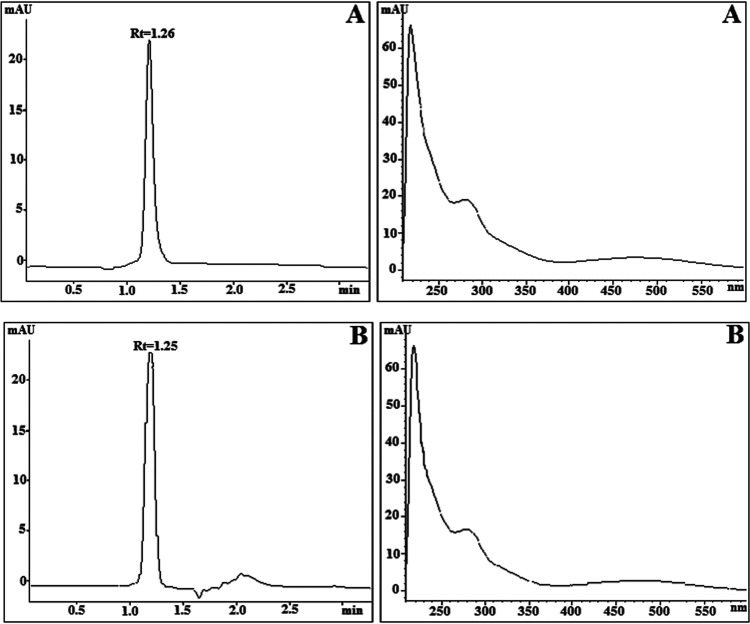
Representative LC-DAD chromatogram and UV–vis spectra obtained at 250 nm from (A) standard solution of BHH/Fe^3+^ at QC_2_ (10 mg/L Fe) and (B) prototype sample (10 mg/L Fe).

The possibility of enhancing the sensitivity (LOD/LOQ) of the method by injecting larger sample volumes (5–20 μL) was considered. The results showed an increase in the signal-to-noise (S/N) ratio when up to 10 μL was injected, above which S/N did not significantly improve and a loss of peak symmetry was evident. Thus, 10 μL was selected as the injection volume. Under the chromatography conditions described above, it was possible to analyze BHH/Fe^3+^ in commercial samples by LC-DAD with an overall run time of 3.5 min (see Figure 4).

### 3.2 MS/MS Confirmation

**Figure 5:**
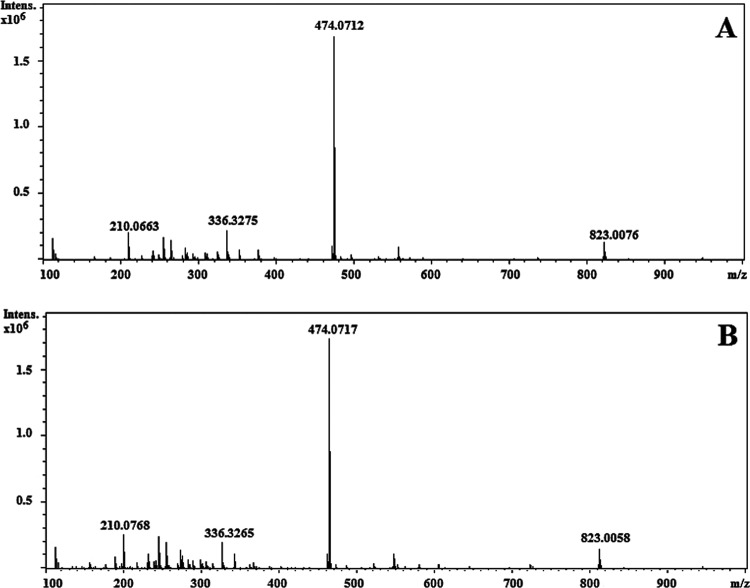
Representative full-scan ESI-MS/MS spectra obtained by direct injection of (A) standard solution (100 μg/L Fe) of BHH/Fe^3+^ and (B) prototype sample (100 μg/L Fe).

To optimize the MS signal, a 2 mg/L solution of BHH/Fe^3+^ was directly injected into the ESI source operated in positive and negative ion modes. Optimal parameter values included negative polarity, capillary voltage of 3400 V, nebulizer pressure of 0.4 bar, drying gas (N_2_) flow of 4 L/min, and a temperature of 200 °C. Figure 5 shows a comparison of the full-scan spectra of a standard solution prepared in the laboratory (Figure 5A) and a commercial prototype sample (Figure 5B). The same signals were obtained in both spectra, showing an intense [M–H]^−^ (precursor ion) corresponding to the molecular ions with the general formula [m*L* + *n*Fe^3+^ – (3*n* + 1)H^+^]^−^, where *L* is the chelating agent (BHH) and *n* is the number of bonded irons. To determine the stoichiometry of complexes (*n* iron/m chelating agent ratio) from *m*/*z*, the characteristic Fe isotopic pattern (^54^Fe/^56^Fe/^57^Fe; 5.9:91.7:2.1), exact mass, and significant fragments (product ions formed by the loss of some neutral molecules) obtained from the precursor ion in the multiple reaction monitoring (MRM) mode to confirm their presence were used. The most representative ion was *m*/*z* 474 [2BHH + Fe^3+^ – 4H^+^], as seen by the isotopic pattern ligand forming a 1:2 (Fe^3+^/BHH) complex, and the transition *m*/*z* 474 → 264, corresponding to a loss of a ligand molecule (C_10_H_12_NO_4_), was used for quantification (complex 1:1). By means of MS/MS data, the ions at *m*/*z* 430 and 210 were identified as product ions and corresponded with the loss of the carboxylate group (CO_2_) from the parent and ligand BHH. ESI-MS/MS spectra and a tentative fragmentation pathway are shown in Figure S3. The synthesis of phenolate-bearing polyaminocarboxylate ligands such as BHH normally leads to the formation of polycondensates of high molecular weight and other byproducts.^26^ The analysis of mass spectra revealed a condensation product at *m*/*z* 830, which was identified as a bromide adduct ion and its fragmentation showed the presence of the complex that can bind three irons following a mono decarboxylation group and loss of water, giving *m*/*z* 747 [3BHH:3Fe^3+^–10H^+^–CO–H_2_O]^−^. The isotopic pattern of the molecular ion at *m*/*z* 747 (100%) and *m*/*z* 745 (19.4%) with the calculated values 100 and 17.7%, respectively, confirmed that the signal at *m*/*z* 747 could be ascribed to the 3:3 stoichiometry and its confirmation ions at *m*/*z* 612 (loss of 2CO_2_ + H_2_O + C_2_H_4_) and *m*/*z* 527 [2BHH:2Fe^3+^–7H]^−^. A free ligand (BHH) was also observed at *m*/*z* 210. Quantification and confirmation transitions are shown in Table 1. Other signals observed in the spectra did not present iron isotopic patterns and were not studied further.

**Table 1:** Characterization of BHH/Fe^3+^ Using MS/MS in Negative Ion Mode

Precursor ion	Product ion (relative intensity)	Collision energy (eV)	Measured *m*/*z* [M–H]^−^	Predicted *m*/*z* [M–H]^−^	Error (ppm)	Molecular formula	Proposed product ion
474.07	210.07(5)^b^	25	210.0773	210.0772	–0.1	C_10_H_12_NO_4_	[BHH]^−^
	263.99(100)^a^	25	263.9957	263.9965	3.1	C_10_H_10_FeNO_4_	[BHH:Fe^3+^–4H^+^−]^−^
	430.08(8)^b^	25	430.0832	430.0833	0.2	C_19_H_22_FeN_2_O_6_	[2BHH:Fe^3+^–4H^+^–CO_2_]^−^
823.00	527.99(13)^b^	30	527.9926	527.9924	–0.3	C_20_H_20_Fe_2_N_2_O_8_	[2BHH:2Fe^3+^–7H]^−^
	612.96(15)^b^	30	612.9696	612.9691	–0.5	C_25_H_23_Fe_3_N_3_O_5_	[3BHH:3Fe^3+^–10H^+^–CO–2H_2_O–C_2_H_4_–2CO_2_]–
	746.99(100)^a^	30	746.9912	746.9907	–0.5	C_29_H_29_Fe_3_N_3_O_10_	[3BHH:3Fe^3+^–10H^+^–CO–H_2_O]^−^

a Product ion used for quantification.

b Product ion used for confirmation.

### 3.3 Method Validation

The method validation was based on the Eurachem Guide^29^ determining the limits of detection and quantification, linearity, precision, and robustness. LOD and LOQ were experimentally determined by measuring the magnitude of the background analytical response at the elution time of BHH/Fe^3+^. LOD and LOQ were estimated as three and ten times the signal-to-noise ratio, and were therefore 0.801 ± 0.0467 and 2.70 ± 0.0413 mg/L Fe, respectively. The use of DAD could be considered a cheap alternative to determine this iron chelate with a high degree of sensitivity and, in the authors’ opinion, it is not necessary to use MS/MS detectors for quantification purposes when high concentrations are expected (50–100 mg/L Fe), as in the determination of Fe-chelates in fertilizers. Working solutions used to construct the calibration curve were prepared using a standard solution over a concentration range of LOQ up to 50 mg/L Fe (calibration levels: LOQ, 5, 10, 25, 50 mg/L Fe). Calibration curves were constructed by plotting the signal on the *y*-axis (analyte peak areas) against the analyte concentration on the *x*-axis and were based on six replicates of each standard solution. The graphs obtained in all of the calibration curves were straight lines, with linearity across the different concentration ranges studied, while the coefficient of the determination values (*R*^2^) was above 0.999. Moreover, the lack of bias was confirmed using a Student’s *t*-test and the distribution of residuals. The precision of the method was evaluated as repeatability (intraday, on the same day, *n* = 6) and intermediate precision (interday, over 3 consecutive days, *n* = 6) as the percentage of relative standard deviation (%RSD) at the three concentrations selected (LOQ, 10, 50 mg/L Fe). Precision was always below 5% (Table S3). These results indicated that the proposed method was precise in accordance with existing norms (%RSD ≤ 20%). Robustness tests were performed to determine the effects presenting small changes in the method parameters as organic mobile-phase composition (30.0 ± 0.5% ACN), pH (8.0 ± 0.5), buffer concentration (0.2 ± 0.05 mM), flow rate (1.00 ± 0.05 mL/min), and detector wavelength (250 ± 0.5 nm). The calculated results, which are given in Table S4, show the robustness of the procedure. The slight changes in the experimental parameters mentioned had no significant effect, confirming the robustness of the method. The storage stability of standard solutions was studied over 2 weeks at different temperatures (−80, −20, 4 °C, and room temperature) protected from the light. The results are given in Table S5. As can be seen, the compound was stable for a short storage time and storage under refrigeration conditions was advisable. Nevertheless, during a long storage time, strong degradation of the compound was observed between 80 and 98% under described storage conditions at all levels. This was more pronounced at room temperature around 98% for all QCs. Therefore, it is recommended that fresh solutions be prepared daily or stored for short periods, no more than 48 h, before analysis by HPLC-DAD.

### 3.4 Application of the Method

**Figure 6:**
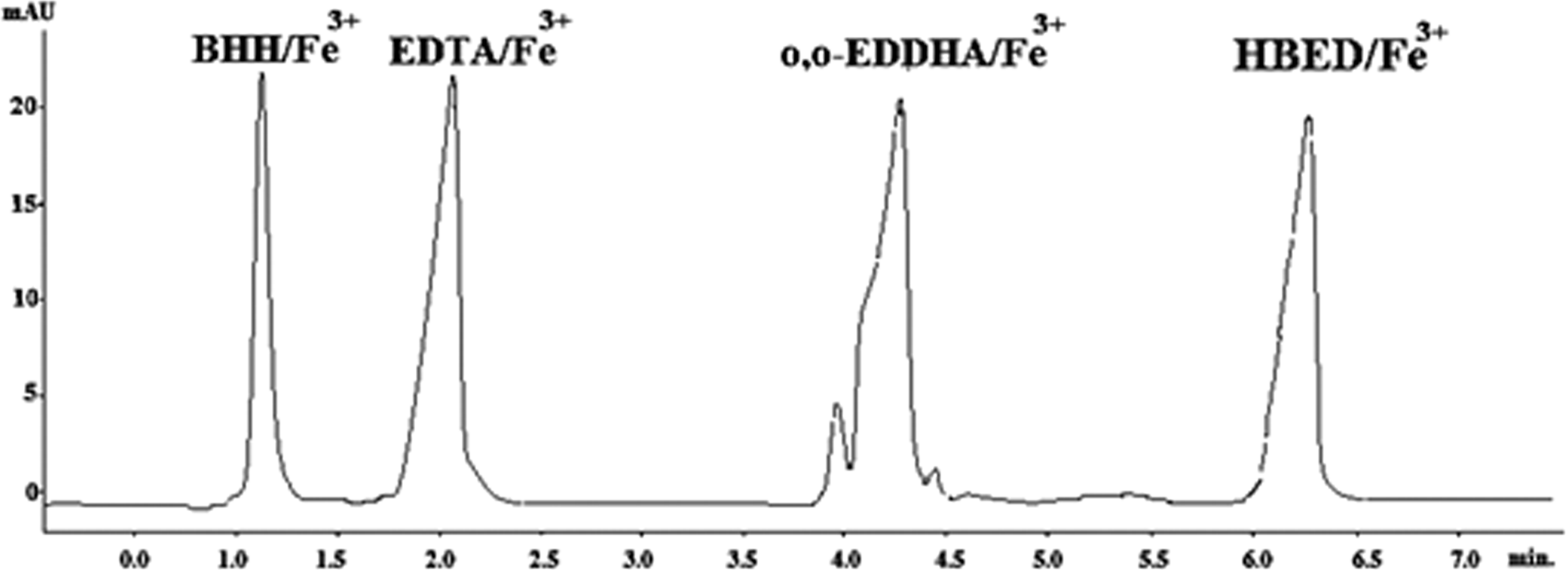
Representative chromatogram of a standard solution of BHH/Fe^3+^, EDTA/Fe^3+^, *o*,*o*-EDDHA/Fe^3+^, and HBED/Fe^3+^ at 10 mg/L.

The validated method was applied to determine BHH/Fe^3+^ in a prototype fertilizer and provided quite similar chromatograms and mass spectra to a standard solution, although some minor differences in ion intensity were observed. A single signal corresponding to a condensation product was detected and identified. No chromatographic interferences were observed at the elution time of the compound in the commercial sample analyzed. The retention times agreed with those previously obtained from the standard solution. The soluble Fe content was measured after digestion, as indicated by EC Regulations 2003/2003 and 1009/2019. The amount of chelated iron in the commercial product was 7.81%. Since the soluble iron in this product was 8%, the chelated fraction (chelated iron with respect to soluble iron) was 98%. These data are in good agreement with the requirements of the regulations cited above where the chelated fraction must be at least 80%. In addition, the presence of other iron chelates that are commonly marketed together as EDTA/Fe^3+^, *o*,*o*-EDDHA/Fe^3+^, or HBED/Fe^3+^ was studied. Figure 6 shows a representative chromatogram of a standard mixture of iron chelates. It was observed that none of the chelates interfere with BHH/Fe^3+^, indicating the selectivity of the method.

In conclusion, this is the first time that an LC-DAD routine method has been developed to determine iron chelated in potential commercial fertilizers containing BHH as the chelating agent for use as a remedy iron chlorosis in calcareous soils. The usefulness of LiChrospher RP-18 was also demonstrated in comparison with other conventional packing materials. The organic modifier, mobile-phase composition, and pH were optimized. The proposed method was fully validated and very good analytical results were obtained, including limit of quantification, a wide range of concentrations, good precision, and robustness. The developed method could be used to quantify the commercial chelate according to the directives regulating this type of product. Moreover, quantification and confirmation transitions were determined by MS/MS and this could be used for further investigations of the dissipation process and potential degradation products of iron chelate in soil. This methodology can be applied to establish a degradation mechanism under environmental conditions as well as the toxicity of degradation products.

## Supporting Information Available

UV–vis spectra of BHH/Fe^3+^ and BHH obtained in a spectrophotometer; peak area of iron chelate obtained after testing the addition of different buffer concentrations in the mobile phase; MS/MS spectrum of BHH/Fe^3+^; physicochemical properties of the analytical columns assayed and a summary of mobile phases tested with Symmetry *C*_18_; and results of precision, robustness, and stability studies (PDF)

## Abbreviations

BHH: benzeneacetic acid, 2-hydroxy-α-[(2-hydroxyethyl)amino]
DEA: diethanolamine
EDA: ethanoldiamine
EDDHA: ethylenediamine-*N*-*N*′bis(*o*-hydroxyphenylacetic)acid
ESI: electrospray ionization source
HILIC: hydrophilic interaction liquid chromatography
MRM: multiple reaction monitoring
QC: quality control
RSD: relative standard deviation
TEA: triethanolamine
