# Supplementary material for: Fast Determination of a Novel Iron Chelate Prototype Used as a Fertilizer by Liquid Chromatography Coupled to a Diode Array Detector
Source: J Agric Food Chem. 2021 Dec 15;69(51):15746–54. doi: 10.1021/acs.jafc.1c05943 (PMC8719335; doi:10.1021/acs.jafc.1c05943)
Supplement: Supplementary file 1 — jf1c05943_si_001.pdf [file jf1c05943_si_001.pdf]

Supplementary material

Fast Determination of a Novel Iron Chelate Prototype Used as Fertilizer by Liquid Lhromatography Coupled to Diode Array Detector

Silvia Valverde*, Alejandra Arcas, Sandra López-Rayo, Juan J.Lucena

Departamento de Química Agrícola y Bromatología. Universidad Autónoma de Madrid, 28049 Madrid, Spain

***Silvia Valverde-** Departamento de Química Agrícola y Bromatología. Universidad Autónoma de Madrid, 28049 Madrid, Spain; orcid.org/0000-0001-9790-7458; Phone +34-914-976518; E-mail: silvia.valverde@uam.es; website: micronutrientsinplants.com

Figure S1. UV-VIS spectrum of **A)** 100 mg/L Fe as BHH/Fe^{3+} standard solution and **B)** 5 mg/L BHH standard solution obtained in a spectrophotometer at pH 8.

Figure S1-A

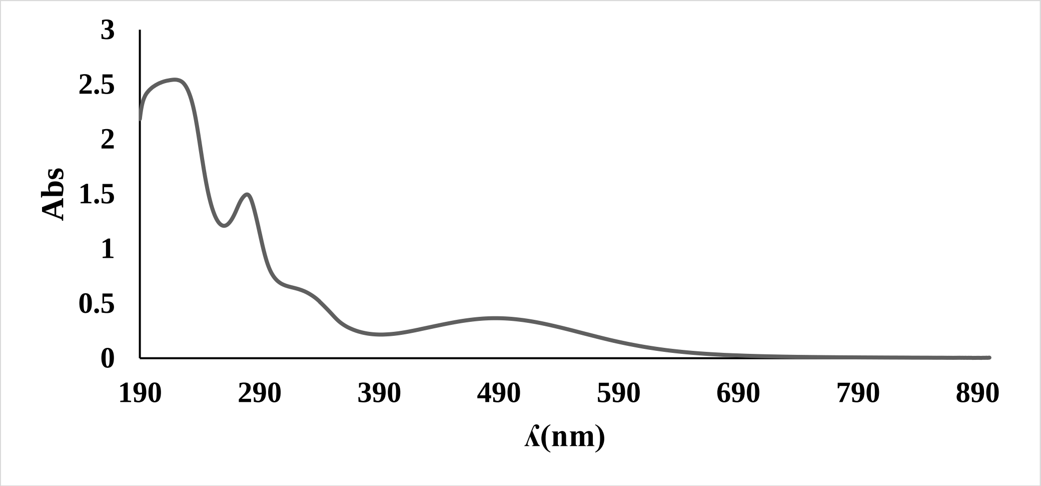

Figure S1-B

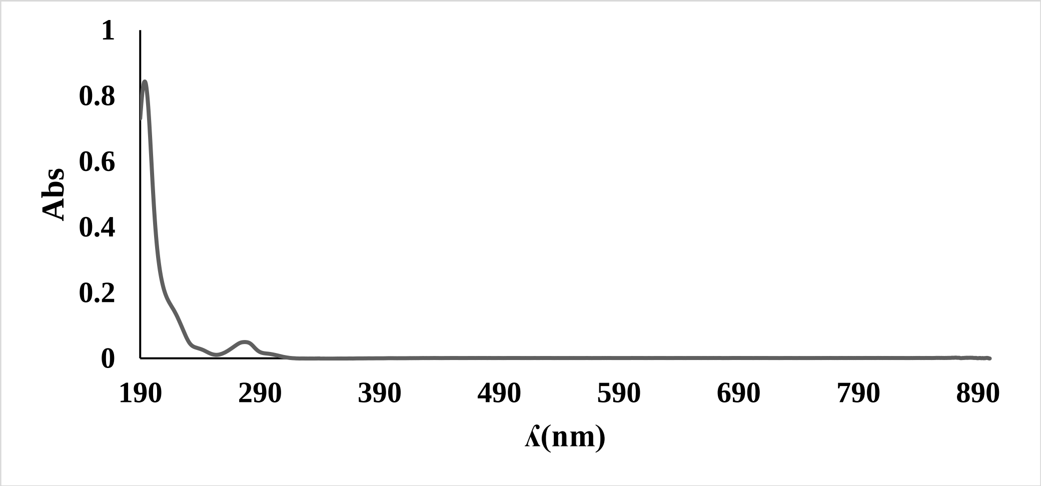

Figure S2. Peak area obtained after testing the addition of different buffer concentrations (n=3) in the mobile phase at the medium QC (10 mg/L Fe).

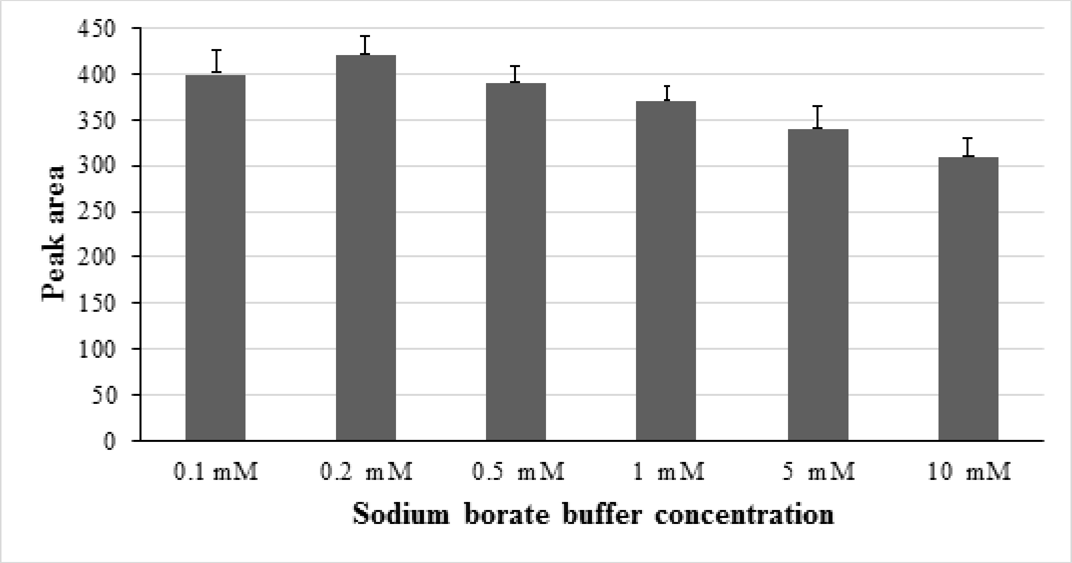

Figure S3. ESI-MS/MS spectrum of m/z 474 obtained by direct injection and proposed fragmentation pathway.

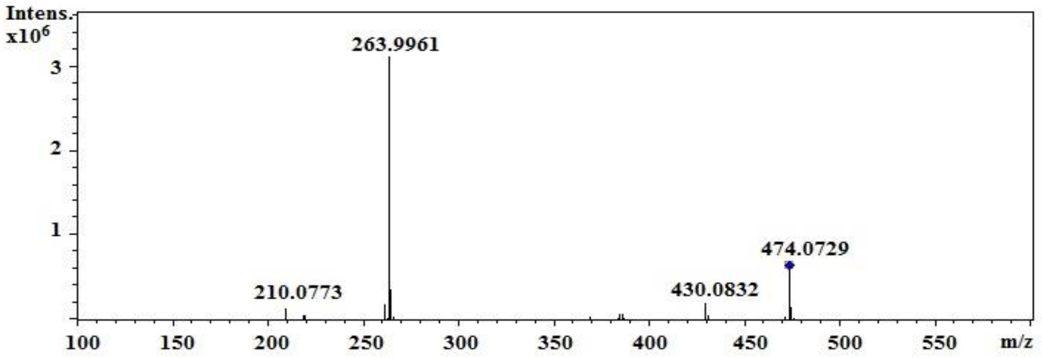

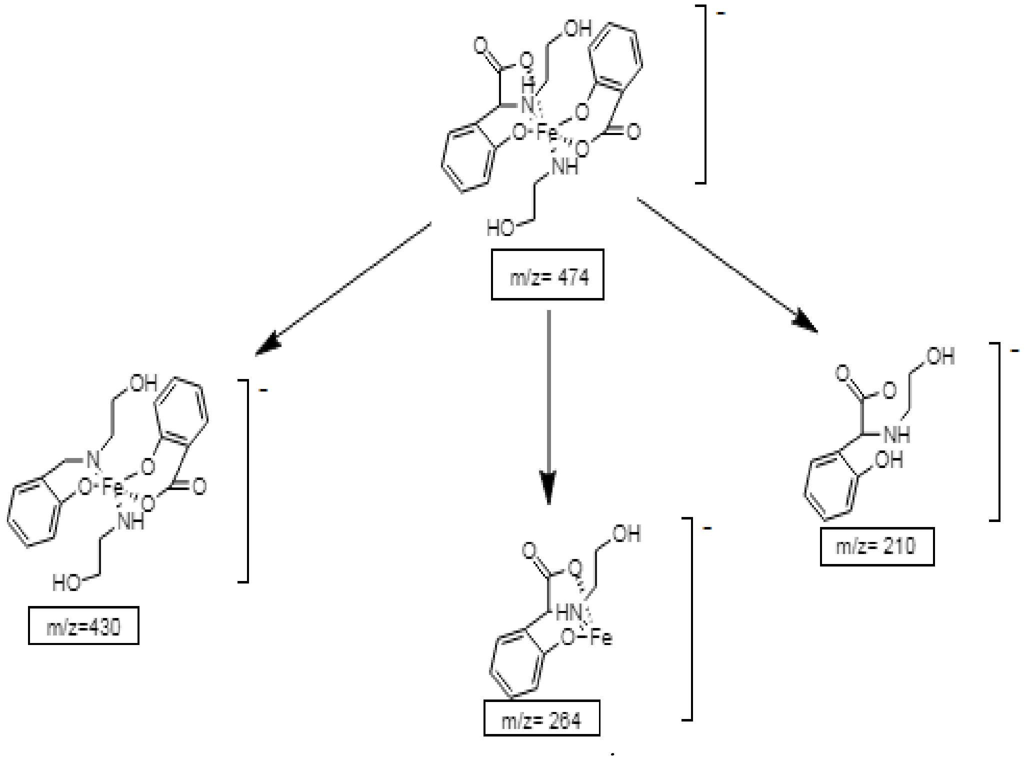

Table S1. Physicochemical properties of the analytical columns assayed.

Analytical column	Particle size (µm)	Pore size (Å)	Surface area (m^2/g)	Total carbon (%)
Symmetry C_{18}	5	100	335	19
Waters Spherisorb ODS2	5	80	220	12
Luna C_{18}	5	100	440	19
LiChrospher RP-18	5	100	350	21
SeQuant ZIC HILIC	3.5	100	180	NS

*NS: not specified

Table S2. A summary of mobile phases tested with Symmetry C_{18} (150 x 4.6 mm, 5 µm).

Test	Organic solvent	Salt	Concentration mM	pH
1	ACN	Phosphate buffer	10	8
2	ACN	Borate buffer	10	8
3	ACN	Ammonium acetate	20	7
4	ACN	Ammonium bicarbonate	10	8
5	ACN	Sodium formate	10	7.5
6	ACN	Tris-HCl	10	8
7	ACN	Trisodium citrate	10	8
8	MeOH	Trisodium citrate	10	8
9	MeOH	Trisodium citrate	50	8

Table S3. Summary of precision studies for determination of BHH (n=6).

	**Intra-day (%RSD)**	**Inter-day (%RSD)**
**QC1**	0.23	0.19
**QC2**	3.2	1.1
**QC3**	1.7	0.73

Table S4. Robustness studies for 10 mg/L Fe as BHH/Fe^{3+} for HPLC-DAD (n=3).

**Parameter**	**Value**	**Retention time (min.)**	**Difference (%)**
**Present study**		1.3 ± 0.0021	
**Flow rate(mL/min)**	0.95	1.3 ± 0.3	105 ± 0.3
	1.05	1.2 ± 0.07	95 ± 0.08
**Percentage of the organic phase (%)**	25	1.3 ± 0.02	103 ± 0.2
	35	1.3 ± 0.1	98 ± 0.09
**Concentration of the buffer (mM)**	0.15	1.2 ± 0.09	94 ± 0.07
	0.25	1.3 ± 0.1	106 ± 0.3
**Detector wavelength (nm)**	245	1.3 ± 0.3	100 ± 0.2
	255	1.3± 0.1	100 ± 0.2

Table S5. Stability for BHH/Fe^{3+} (n=6).

Storage	Quality control at 0h		Short-term stability (24 h)	Short-term stability (48 h)	Long-term stability (2 weeks)
**Room temperature**	QC_1 ± RSD (%)	119 ± 0.1	100 ± 0.2	89 ± 5.0	2.00±1.0
	QC_2± RSD (%)	405 ± 0.6	389 ± 3.2	408± 0.2	10±4.0
	QC_3± RSD (%)	2001 ± 1.0	1976 ± 1.7	2113± 1.7	48±8.0
**4 ºC**	QC_1± RSD (%)		101 ± 0.9	97 ± 1.6	12±2.3
	QC_2± RSD (%)		394 ± 0.2	395± 0.5	23±5.0
	QC_3± RSD (%)		1999 ± 0.7	2094± 3.4	64±5.0
**-20 ºC**	QC_1± RSD (%)		104 ± 1.0	105 ± 4.4	19±4.0
	QC_2± RSD (%)		401 ± 1.3	402± 0.6	31±1.0
	QC_3± RSD (%)		2034 ± 1.8	2112± 1.7	84±7.0
**-80 ºC**	QC_1± RSD (%)		100 ± 0.6	102 ± 0.8	14±3.7
	QC_2± RSD (%)		396 ± 0.7	397± 0.3	26±1.3
	QC_3± RSD (%)		2002 ± 0.5	1989± 0.1	73±4.5
